# Acrodysostosis type 1: mechanisms explaining PRKAR1A mutation mediated dysregulation of cAMP-PKA signalling

**DOI:** 10.1186/s12964-026-02890-4

**Published:** 2026-05-08

**Authors:** Harry Moxom, Susan J. Kimber

**Affiliations:** Division of Cell Matrix Biology and Regenerative Medicine, School of Biological Sciences. Faculty of Biology Medicine and Health, University of Manchester, M13 9PT Manchester, UK

**Keywords:** PRKAR1A, cAMP, Protein kinase A, Acrodysostosis, Carney complex, Signal transduction, Dominant-negative mechanism, Endocrine resistance

## Abstract

Acrodysostosis type 1 (ACRDYS1) is a rare multisystem developmental disorder affecting skeletal growth, endocrine function, neurodevelopment, metabolism, and tooth formation. It is caused by heterozygous mutations in PRKAR1A, which encodes the type Iα regulatory subunit (RIα) of protein kinase A (PKA), a central mediator of cyclic AMP (cAMP)-dependent signalling. Although ACRDYS1 belongs to the broader family of Gsα-cAMP-PKA-related disorders, its underlying mechanism is distinct. Disease-associated PRKAR1A mutations cluster within regions of RIα that bind cAMP and undergo conformational rearrangements required for PKA activation. These variants impair cAMP binding and disrupt the structural transitions needed to disinhibit catalytic subunits. Importantly, mutant RIα is expressed at near-normal levels and assembles efficiently into PKA holoenzymes, but these complexes respond weakly and sluggishly to physiological cAMP signals. Drawing on structural, biochemical, cellular, and in vivo studies, we define a dual pathogenic mechanism underlying ACRDYS1. First, defective cAMP-driven conformational changes reduce the sensitivity and amplitude of type I PKA activation, producing a hypomorphic signalling state despite intact upstream receptor coupling and cAMP production. Second, activation-resistant RIα holoenzymes impose a dominant-negative constraint by retaining catalytic subunits, further limiting the pool available for productive signalling in heterozygous cells. We relate this core defect in signal responsiveness to tissue-specific vulnerability. Impaired RIα-dependent decoding of cAMP signals disrupts the Ihh-PTHrP feedback loop in the growth plate, blunts hormone-responsive transcriptional programmes in endocrine epithelia, and alters spatially restricted PKA signalling domains in neurons and metabolically active tissues. Despite the diversity of affected organs, the unifying defect is an inability to generate appropriately timed and scaled PKA responses. This framework establishes ACRDYS1 as a disorder of signal decoding rather than signal generation, clarifies its mechanistic distinction from PRKAR1A-related Carney Complex, and highlights therapeutic strategies aimed at restoring local cAMP–PKA signalling dynamics rather than globally amplifying pathway activity.

## Introduction

Protein kinase A (PKA) is a central effector of cyclic adenosine monophosphate (cAMP) signalling and governs diverse cellular processes including proliferation, differentiation, endocrine responses and metabolic regulation [1–3]. The fidelity of PKA activation depends on the precise allosteric behaviour of its regulatory subunits, which bind cAMP and control inhibition status of the catalytic subunits [2–4]. Among the four regulatory genes, RIα, encoded by *PRKAR1A*, is uniquely positioned as the dominant regulator of rapid, transient cAMP responses in the majority of developmental contexts [2, 5, 6]. Acrodysostosis type 1 (ACRDYS1) is a rare multisystem, developmental disorder caused by heterozygous mutations in *PRKAR1A* that selectively impair the activation of RIα-containing PKA holoenzymes [7–9]. Clinically, ACRDYS1 is characterised by short stature, severe brachydactyly, craniofacial abnormalities, advanced bone age and multihormone resistance, often accompanied by neurodevelopmental and metabolic disturbances [7–10]. The phenotype reflects a unifying molecular defect: pathological RIα variants with defective cAMP binding and impaired allosteric transitions. The resultant incomplete disinhibition of catalytic subunits leads to blunted PKA activation despite intact upstream signalling [11–13]. Although ACRDYS1 shares phenotypic overlap with other disorders of the Gsα–cAMP–PKA axis, such as pseudohypoparathyroidism, PDE4D-related Acrodysostosis, and Carney Complex [9, 14], its pathology is mechanistically distinct. This review therefore centres specifically on how disease-associated PRKAR1A mutations disrupt RIα structure and regulatory behaviour, and how these biochemical defects propagate through tissue-specific signalling networks to generate the characteristic ACRDYS1 phenotype [6, 15]. Drawing on structural, biochemical, cellular and in vivo evidence, we define the core molecular consequences of PRKAR1A dysfunction; reduced cAMP binding, impaired RIα conformational switching, and persistent retention of catalytic subunits [11–13]; and relate these defects to the major systems affected in ACRDYS1, including skeletal development, endocrine signalling, neurodevelopment and metabolism [7–10, 16], illustrating how disruption of a single regulatory node within the cAMP–PKA axis can produce a multisystem phenotype.

### Protein kinase A: structure, function and regulation

#### PKA holoenzyme architecture

**Fig. 1:**
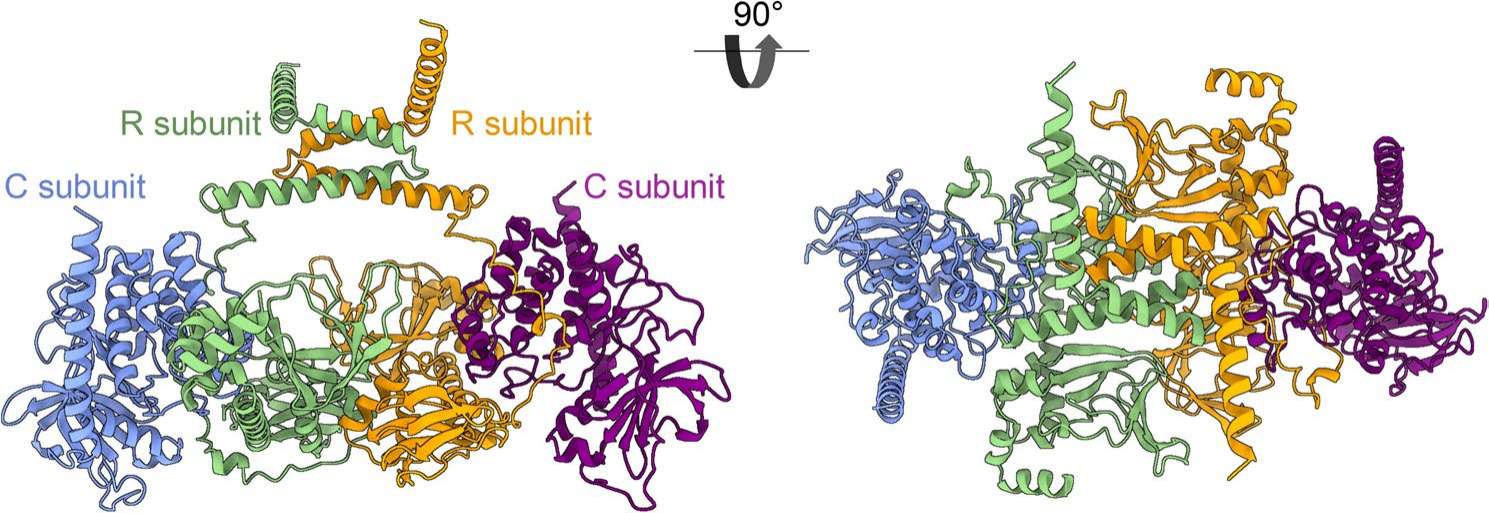
Architecture of the inactive PKA holoenzyme (R₂C₂) and regulatory–catalytic subunit organisation. Ribbon representations of the cAMP-free protein kinase A (PKA) holoenzyme composed of a dimer of regulatory subunits (R) bound to two catalytic subunits (C). The two regulatory subunits (RIα) are shown in green and orange, and the two catalytic subunits are shown in blue and purple. The left panel shows a frontal view highlighting the overall R-C arrangement, while the right panel shows the same complex rotated (90^0^) to emphasise the extended architecture and relative positioning of the catalytic subunits with respect to the regulatory dimer

PKA typically exists as an inactive heterotetrameric holoenzyme (R₂C₂) composed of two regulatory (R) and two catalytic (C) subunits [2, 17, 18] (Fig. 1). In the basal state, an inhibitory segment within each regulatory subunit occupies the catalytic cleft of the paired C subunit, preventing kinase activity until appropriate cAMP signals are received [18–20]. This architectural arrangement ensures that PKA activation is tightly coupled to second-messenger dynamics [2, 18, 21].

### Regulatory subunit genes and their mechanistic relevance to ACRDYS1

Four genes encode the PKA regulatory subunits, PRKAR1A (RIα), PRKAR1B (RIβ), PRKAR2A (RIIα) and PRKAR2B (RIIβ), which combine with catalytic subunits to form type I or type II holoenzymes [2, 22, 23]. Although these PKA regulatory genes share a conserved domain architecture and are generally expressed in all cell types, they differ markedly in activation properties, anchoring behaviour and the ratio at which they are represented in different tissues [2, 22, 24]. A key determinant of these differences is the spatial organisation of PKA signalling through A-kinase anchoring proteins (AKAPs), which tether PKA holoenzymes to defined subcellular microdomains and thereby shape the localisation and specificity of cAMP responses [24–26]. RIα-containing holoenzymes respond to cAMP with low activation thresholds and rapid kinetics, making them particularly suited to contexts requiring fast, transient signalling [27–29]. Across human developmental transcriptomic datasets, PRKAR1A is widely expressed and frequently the dominant regulatory gene in chondrogenic mesenchyme, growth plate cartilage, thyroid epithelium and adrenal tissues, all of which rely heavily on dynamic cAMP-PKA responses to regulate proliferation, and hormone responses [7–9, 30]. By contrast, PRKAR1B exhibits a more restricted expression pattern and is enriched within specific neuronal lineages, including cortical and hippocampal neurons, where it supports aspects of synaptic maturation and activity-dependent transcription [22, 31, 32]. RIIα and RIIβ, meanwhile, are preferentially expressed in tissues with strong AKAP-dependent scaffolding requirements, such as cardiac muscle, mature adipose depots and subsets of differentiated neurons [24, 25, 33]. Their tight anchoring to AKAPs positions type II holoenzymes within spatially restricted microdomains that respond to sustained, often compartmentalised cAMP signals rather than the rapid fluctuations that are typically sensed by RIα [24–26].

This regulatory architecture has direct implications for ACRDYS1. Because disease-associated PRKAR1A variants impair the activation mechanics of RIα, tissues that physiologically depend more strongly on type I PKA signalling are selectively vulnerable despite the ubiquitous expression of PRKAR1A [7–9, 12]. Conversely, tissues in which RIβ or RII PKA complexes dominate may retain more intact PKA signalling because their activation does not rely primarily on RIα [22, 24, 25]. This principle of regulatory subunit dependence helps explain the tissue-selective phenotype of ACRDYS1 and reinforces the central theme of this review: that the disease reflects disruption of RIα-dependent signal decoding rather than a global failure of the PKA pathway [7–9, 12].

### Domain architecture of RIα

**Fig. 2:**
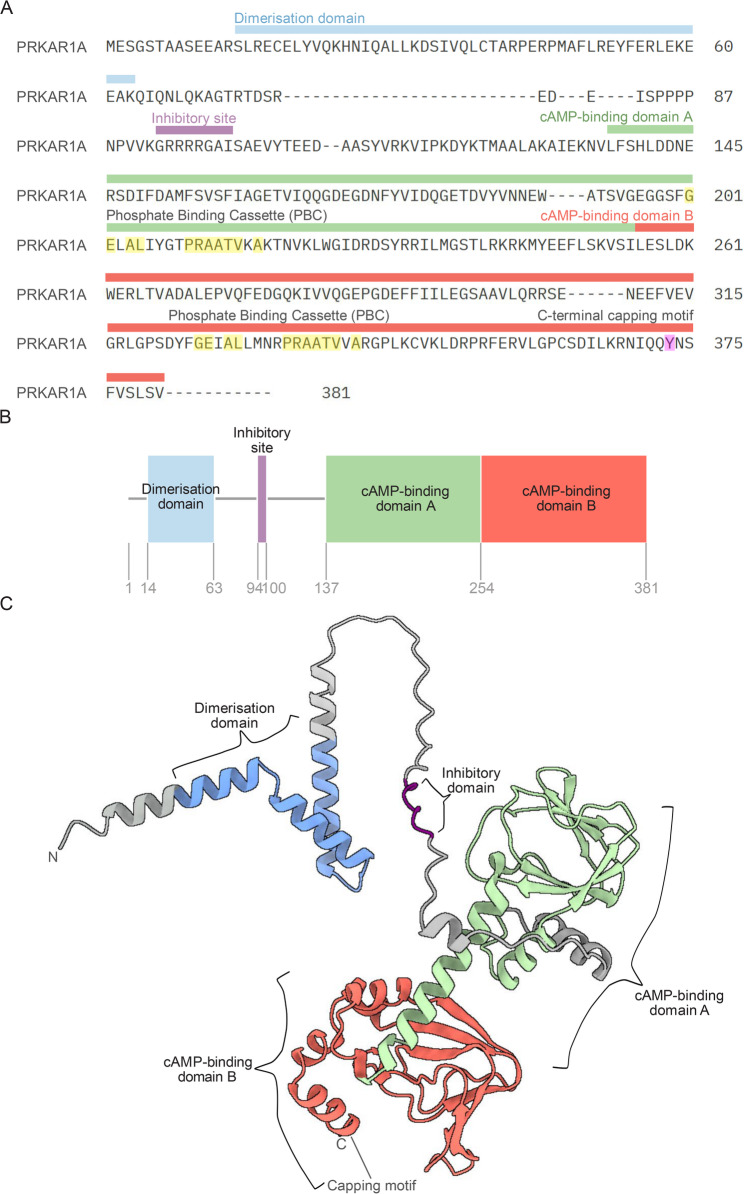
Domain architecture and structural organisation of the PKA RIα regulatory subunit. **A **Annotated primary sequence of the human PRKAR1A (RIα) protein showing the location of major structural and functional domains along the polypeptide chain. The N-terminal dimerisation/docking domain is shown in blue, followed by the inhibitory site in purple. The two C-terminal cyclic nucleotide–binding domains, CNB-A (green) and CNB-B (red), are indicated. Within the CNB domains, conserved phosphate-binding cassette (PBC) motifs are highlighted in yellow, and the C-terminal capping motif is highlighted in pink. Residue numbers are shown at the right of each sequence segment. **B **Simplified linear schematic of RIα summarising the relative positions and approximate residue boundaries of the dimerisation/docking domain, inhibitory site, CNB-A and CNB-B domains, corresponding to the annotated sequence in panel A. **C **Ribbon representation of RIα illustrating the three-dimensional spatial arrangement of these domains. The extended N-terminal dimerisation/docking domain, inhibitory segment, and the two C-terminal CNB domains are coloured consistently with panels A and B

Despite their gene-specific functional roles, all regulatory subunits share a conserved modular architecture [2, 17, 18]. The N-terminal dimerisation/docking (D/D) domain mediates regulatory–regulatory pairing and interaction with AKAPs [17, 24, 34] (Fig. 2A, B). Immediately downstream lies the inhibitory segment (Fig. 2A), which in RI genes functions as a pseudosubstrate that occupies the catalytic cleft of each associated C subunit [18, 20, 35]. The C-terminal half of the protein contains two tandem cyclic nucleotide-binding domains, CNB-A and CNB-B, which are arranged sequentially along the primary sequence (Fig. 2A, B) and adopt compact β-barrel folds capped by C-terminal α-helical elements (Fig. 2C) [18, 19, 36]. Both domains contain highly conserved phosphate-binding cassettes (PBCs), characterised by the consensus G-E-X-A-L-X₄-P-R-A-A-T-V-X-A motif, which are highlighted within the sequence of Fig. 2A and coordinate the cyclic phosphate to define nucleotide affinity [19, 36, 37]. cAMP binds the two CNB domains sequentially and cooperatively. CNB-B engages cAMP first, aided by its greater complement of positively charged residues within the PBC, and this initial binding induces a hinge-mediated rearrangement that brings the two CNB domains into closer proximity [27, 29, 38] (Fig. 2C). This conformational priming facilitates subsequent cAMP binding to CNB-A, and the ordered occupation of both domains drives the allosteric transitions required for disinhibition of the catalytic subunits [18, 27, 29]. In RIα, the CNB-B domain contains an additional C-terminal capping motif, Tyr373, (Fig. 2A) and forms part of the C-terminal helical structure that holds the cAMP molecule in place in CNB-B’s binding pocket (Fig. 2C) [11, 38, 39]. Subtle perturbations to the geometry of the PBC, β-barrel scaffold or capping region can therefore markedly shift the energetic landscape of activation [11, 18, 38]. It is within these conserved structural elements that most ACRDYS1-associated PRKAR1A mutations occur, rendering the RIα scaffold uniquely susceptible to pathological disruption [7–9, 11].

## cAMP-PKA signalling pathway

**Fig. 3:**
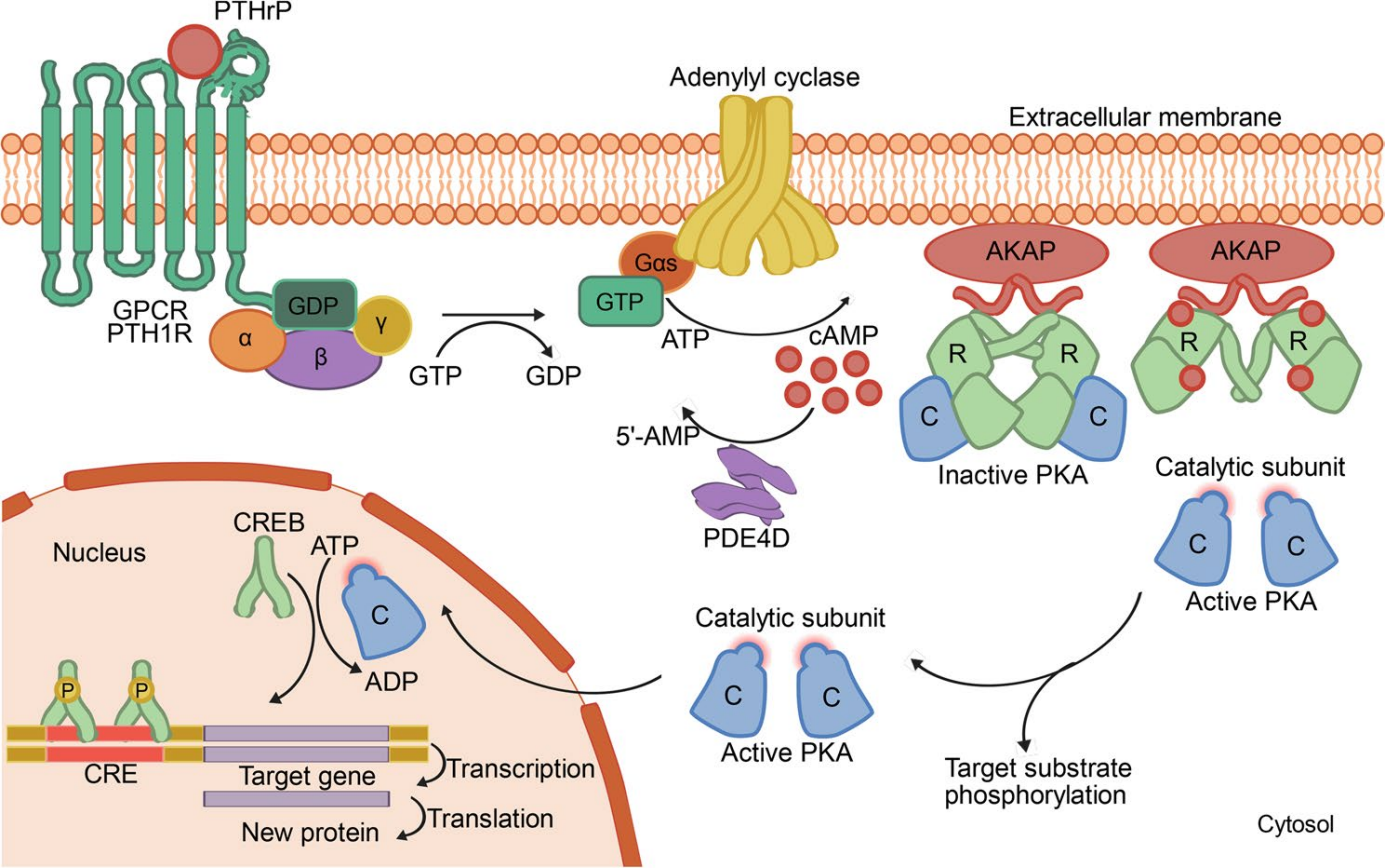
Canonical Gs–cAMP–PKA signalling pathway and transcriptional output. Schematic overview of cAMP-dependent PKA signalling following activation of a Gs-coupled G protein–coupled receptor (GPCR). Ligand binding promotes GDP–GTP exchange on the Gsα subunit, which stimulates adenylate cyclase to generate cAMP from ATP. cAMP accumulates within local signalling microdomains shaped by phosphodiesterases and A-kinase anchoring proteins (AKAPs), which scaffold PKA holoenzymes in proximity to receptors and downstream substrates. Binding of cAMP to the regulatory (R) subunits of PKA induces dissociation of the catalytic (C) subunits, enabling phosphorylation of cytoplasmic targets and translocation of a fraction of active C subunits to the nucleus. In the nucleus, catalytic subunits phosphorylate the transcription factor CREB, promoting recruitment of coactivators to cAMP response elements (CREs) and induction of target gene transcription

cAMP signalling serves as the principal mechanism by which Gs-coupled receptors translate extracellular hormonal cues into intracellular kinase activity [21, 30, 40]. Activation begins when ligands such as PTH, TSH or ACTH bind their respective receptors, PTH1R, TSHR and ACTHR, promoting GDP–GTP exchange on the Gsα subunit [21, 41, 42] (Fig. 3). Activated Gsα–GTP stimulates membrane-bound adenylate cyclase, catalysing the conversion of ATP into cAMP [21, 30, 40]. This rise in cAMP does not diffuse uniformly throughout the cytoplasm but spreads through permissive microdomains [15, 30, 43]. These microdomains are shaped by the local interactions of phosphodiesterases, such as PDE4D, which hydrolyses cAMP to 5′-AMP, and AKAPs (Fig. 3), which scaffold adenylate cyclases, PDEs, PKA holoenzymes and downstream substrates into spatially discrete signalling compartments [15, 30, 43]. Within these microdomains, cAMP interacts with PKA holoenzymes, initiating the conformational cycle that governs catalytic disinhibition [2, 18, 27]. Activation of PKA begins when cAMP binds the CNB-B domain of each regulatory subunit, inducing an initial rearrangement of the CNB-B and CNB-A domains [27, 29, 38] (Fig. 3). This primed configuration and cooperative occupation of both domains drive closure of the α-helical lids, destabilisation of the inhibitory contact and eventual disinhibition of the catalytic subunits [18, 27, 29].

Once activated, PKA catalytic subunits phosphorylate a diverse set of cytoplasmic targets, including ion channels such as the cystic fibrosis transmembrane conductance regulator (CFTR) and L-type Ca²⁺ channels, metabolic enzymes such as phosphorylase kinase and hormone-sensitive lipase, and cytoskeletal regulators including vasodilator-stimulated phosphoprotein (VASP) and the small GTPase RhoA [1, 2, 44]. A fraction of these catalytic subunits translocate to the nucleus, where they phosphorylate transcription factors such as cyclic AMP response element-binding protein (CREB), ATF1 and CREM [23, 24, 27] (Fig. 3). In the example of CREB, once phosphorylated, CREB recruits the coactivators CREB-binding protein (CBP) and p300 to initiate chromatin remodelling and promote transcription of cAMP-responsive genes, including those regulating cell cycle progression, extracellular matrix remodelling, endocrine responsiveness and developmental patterning [23, 24, 27]. The amplitude and duration of this transcriptional response depend on the efficiency of catalytic disinhibition and the integrity of RIα-mediated activation [18, 23, 27].

It is at the stage of the PKA activation cycle, not at the level of receptor signalling, Gs activation, adenylate cyclase function or cAMP production, that ACRDYS1 arises. Mutations in PRKAR1A disrupt the ability of RIα to bind cAMP with appropriate affinity or undergo the conformational transitions required for catalytic disinhibition [38, 45, 46]. Although upstream signalling remains intact, the holoenzyme fails to respond normally to physiological cAMP increases, resulting in attenuated activation of catalytic subunits, reduced phosphorylation of CREB and a blunted transcriptional programme [23, 38, 45]. Tissues that depend predominantly on type I PKA are therefore disproportionately affected [2, 7, 38]. In this way, the pathogenesis of ACRDYS1 reflects a selective defect in the molecular machinery of PKA activation rather than a perturbation of cAMP synthesis or degradation [7, 30, 38].

### PRKAR1A mutation clusters in ACRDYS1

**Fig. 4:**
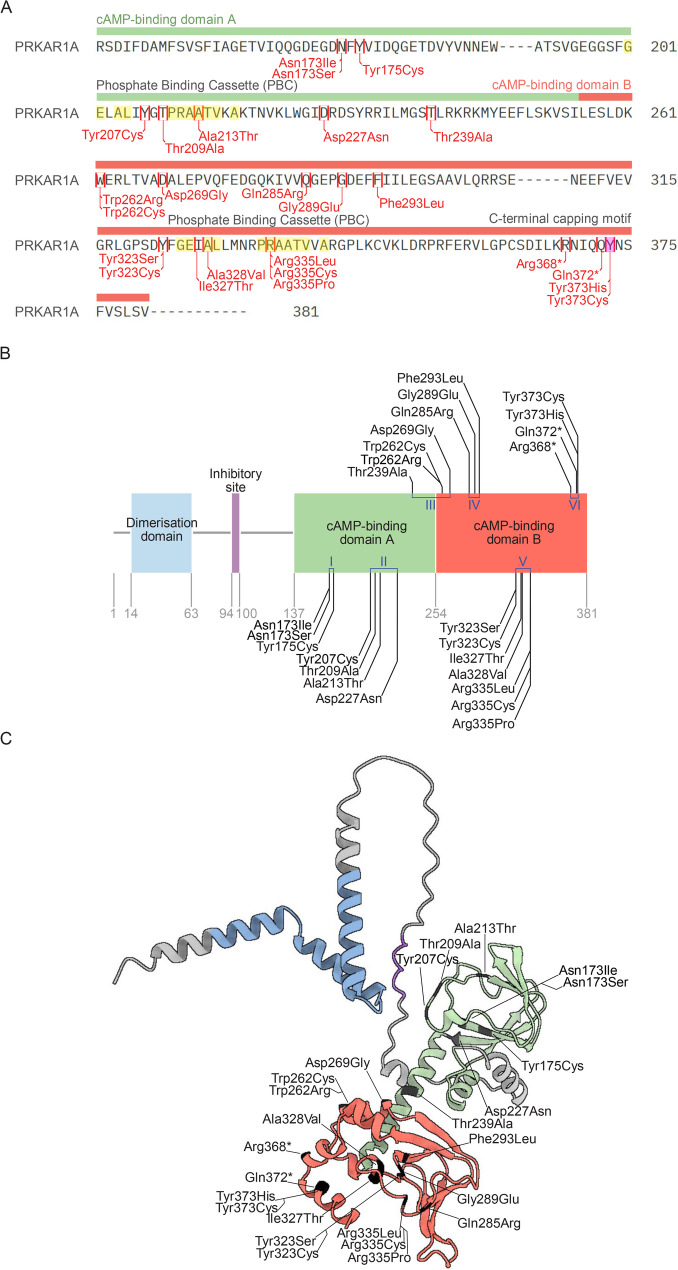
Clustering of ACRDYS1–associated PRKAR1A mutations within the RIα cyclic nucleotide-binding domains. **A **Annotated primary sequence of the human PRKAR1A (RIα) protein showing the positions of amino acid substitutions and truncating variants reported in ACRDYS1. Mutations are indicated in red and mapped onto the two cyclic nucleotide-binding domains, CNB-A (green) and CNB-B (red). Conserved phosphate-binding cassette (PBC) motifs within each CNB domain are highlighted in yellow, and the C-terminal capping motif is highlighted in pink. Residue numbers are shown at the right of each sequence segment. **B **Simplified linear schematic of RIα summarising the relative positions of the dimerisation/docking domain, inhibitory site, CNB-A and CNB-B domains, with Acrodysostosis-associated mutations mapped onto the corresponding regions of the protein. Roman numerals indicate the mutation groupings referenced in the text. **C **Ribbon representation of RIα illustrating the three-dimensional spatial distribution of ACRDYS1–associated mutations. Residues affected by disease-associated variants are labelled and shown within the folded CNB-A and CNB-B domains, including positions within β-barrel cores, phosphate-binding cassette regions, interdomain interface regions and the C-terminal capping motif. Domains are coloured consistently with panels A and B

Pathogenic PRKAR1A variants associated with ACRDYS1 cluster exclusively within the two cyclic nucleotide-binding domains of RIα (Fig. 4A; Table 1). Among currently reported patient cohorts, no ACRDYS1-causing mutations occur within the dimerisation domain or the inhibitory sequence, both of which remain structurally and functionally intact in ACRDYS1 [38, 47]. Within the CNB domains, missense variants localise to residues that shape the CNB-A and CNB-B pockets, stabilise their β-barrel scaffolds, mediate interdomain communication or secure the active conformation of the protein [20, 38, 47]. Together, this distribution supports the view that the disorder primarily arises from disruption of the allosteric transitions required for cAMP-dependent activation. However, because ACRDYS1 is extremely rare and remains under-studied, secondary effects on holoenzyme stability, assembly dynamics, or subcellular compartmentalisation cannot be excluded [4, 38, 45]. Although structurally diverse, these mutations fall into several mechanistically coherent clusters [20, 38, 47] (Fig. 4B).

**Table 1:** Reported PRKAR1A variants causing ACRDYS1. References correspond to the first published report identifying each PRKAR1A variant in individuals with ACRDYS1

Amino acid change:	Reported in:
p.Asn173Ile	Ertl et al. 2023 [48]
p.Asn173Ser	Ertl et al. 2023 [48]
p.Tyr175Cys	Elli et al. 2016 [14]
p.Tyr207Cys	Ertl et al. 2023 [48]
p.Thr209Ala	Elli et al. 2016 [14]
p.Ala213Thr	Mühn et al. 2013 [49]
p.Asp227Asn	Mühn et al. 2013 [49]
p.Thr239Ala	Nagasaki et al. 2012 [50]
p.Trp262Arg	Michot et al. 2018 [51]
p.Trp262Cys	Ertl et al. 2023 [48]
p.Asp269Gly	Elli et al. 2016 [14]
p.Gln285Arg	Linglart et al. 2012 [8]
p.Gly289Glu	Linglart et al. 2012 [8]; Li et al. 2014 [52]
p.Phe293Leu	Elli et al. 2016 [14]
p.Tyr323Cys	Michot et al. 2018 [51]
p.Tyr323Ser	Michot et al. 2018 [51]
p.Ile327Thr	Lee et al. 2012 [53]
p.Ala328Val	Linglart et al. 2012 [8]
p.Arg335Leu	Linglart et al. 2012 [8]
p.Arg335Cys	Mühn et al. 2013 [49]
p.Arg335Pro	Lee et al. 2012 [53]
p.Arg368X	Mühn et al. 2013 [49]; Linglart et al. 2011 [7]; Kaname et al. 2014 [48]
p.Gln372X	Linglart et al. 2012 [8]
p.Tyr373His	Michot et al. 2018 [51]
p.Tyr373Cys	Mühn et al. 2013 [49]

### Cluster I - CNB-A structural core

The first cluster comprises variants such as p.Asn173Ile, p.Asn173Ser and p.Tyr175Cys, which affect residues that stabilise the CNB-A β-barrel and the geometry of the phosphate-binding cassette rather than contacting cAMP directly [20, 47] (Fig. 4.A, C). These substitutions are theorised to alter the rigidity of the CNB-A core, raising the energetic barrier required for lid closure and catalytic disinhibition [20, 47]. Biochemically, they do not disrupt protein expression and holoenzyme assembly but shift cAMP dose-response curves to the right and reduce maximal activation, reflecting impaired structural transitions rather than diminished cAMP affinity [20, 47].

### Cluster II - CNB-A pocket geometry and PBC hydrogen-bonding network

The second group of mutations: p.Tyr207Cys, p.Thr209Ala, p.Ala213Thr, p.Asp227Asn and p.Thr239Ala, affect residues that line the CNB-A binding pocket or participate in the hydrogen-bonding network required to stabilise cAMP [20, 47] (Fig. 4.A, C). These variants are suspected to distort the pocket geometry or disrupt the PBC interaction network, lowering cAMP affinity and reducing the efficiency of cAMP-induced dissociation [20, 47]. This “pocket distortion” mechanism produces an incomplete catalytic activation even under conditions where CNB-B remains functional [20, 47].

### Cluster III - hinge region and allosteric transmission

A third cluster comprises residues located within the hinge region connecting the CNB-A and CNB-B domains, including variants such as p.Trp262Arg, p.Trp262Cys and p.Asp269Gly [20, 38, 47] (Fig. 4.A–C). This segment functions as the primary mechanical pivot that enables coordinated movement between the two cyclic-nucleotide binding domains during activation. These residues do not primarily determine cAMP affinity but instead mediate the transmission of conformational changes initiated in CNB-B to CNB-A and onward to the inhibitory interface [18, 20, 38] (Fig. 4.C). Variants in this region often bind cAMP with near-normal affinity yet fail to propagate the structural rearrangements required for catalytic disinhibition. Their biochemical profile therefore reflects an allosteric uncoupling phenotype, in which the holoenzyme senses cAMP but remains locked in an inhibited state [20, 35, 47].

### Cluster IV - interdomain transition and CNB-A/CNB-B coupling

A fourth cluster maps to residues positioned at the structural transition between the CNB-A and CNB-B domains, including p.Gln285Arg, p.Gly289Glu and p.Phe293Leu [20, 38, 47] (Fig. 4A-C). This region lies immediately adjacent to the hinge described above but forms part of the interdomain transition module that stabilises the relative orientation of the two cyclic-nucleotide binding domains during activation. Whereas hinge residues primarily transmit conformational changes between domains, substitutions in this neighbouring segment disrupt the structural rearrangements that organise CNB-B following cAMP-induced movements. Consequently, these variants often retain near-normal cAMP binding yet fail to support efficient translation of the activation signal, leading to incomplete catalytic disinhibition despite intact holoenzyme assembly [20, 38, 47].

### Cluster V - CNB-B pocket and interdomain cooperativity

Further mutations localise to the CNB-B domain, including p.Tyr323Ser, p.Tyr323Cys, p.Ile327Thr, p.Ala328Val and p.Arg335Lys/Cys/Pro substitutions [20, 47] (Fig. 4.A, C). These residues contribute to the primary cAMP-binding pocket [27, 38, 47]. Because CNB-B is the initial site of cAMP engagement that primes the activation cycle, these variants reduce the sensitivity of RIα to physiological cAMP levels [27, 47]. The resulting activation curves are shallow and incomplete even at high cAMP concentrations, indicating that initiation of the activation sequence is impaired [20, 47].

### Cluster VI - C-terminal capping region and open-state stability

A final cluster affects the C-terminal capping region of CNB-B, encompassing variants such as p.Arg368X, p.Gln372X, p.Tyr373His and p.Tyr373Cys [20, 34, 47] (Fig. 4.A, B, C). These residues stabilise the adenine ring of cAMP and secure the CNB-B domain in its active conformation [20, 47]. Loss or alteration of this hydrophobic cap destabilises cAMP binding and prevents the domain from achieving the closed conformation required for catalytic disinhibition [20, 34, 47]. Structural analyses of the most studied ACRDYS1 PRKAR1A variant, p.Arg368X, shows complete loss of the capping motif, resulting in persistent association of the catalytic subunit and severely impaired activation [20]. Knock-in mice harbouring p.R368X recapitulate skeletal and endocrine abnormalities characteristic of ACRDYS1 suggested to be the result of reduced dissociation of PKA causing reduced cAMP signalling, validating this mechanism in vivo [34].

Across all six clusters, a unifying mechanistic theme emerges: each mutation disrupts a distinct aspect of the cAMP-driven activation cycle, whether through altered initial engagement in CNB-B, reduced affinity or pocket integrity in CNB-A, impaired interdomain coupling via the hinge, or failure to stabilise the active conformation at the capping region [20, 38, 47]. Despite acting at different structural points, the collective biochemical consequence is the same: RIα becomes less responsive to physiological cAMP elevations and disinhibits catalytic subunits inefficiently [20, 34, 47]. This attenuated sensitivity to cAMP, combined with reduced catalytic disinhibition, produces a reduced signalling response characteristic of ACRDYS1 [20, 38, 47]. Importantly, because holoenzyme formation remains intact, these activation-resistant RIα variants can still bind catalytic subunits but fail to disinhibit them efficiently [20, 35, 47]. It can therefore be speculated that this may create the conditions for an additional major pathological mechanism in ACRDYS1: accumulative retention of catalytic subunits [34, 35, 47].

### Retention of catalytic subunits in ACRDYS1: experimental evidence and mechanistic framework

Impaired cAMP responsiveness of RIα is a consistent feature of ACRDYS1, but this defect alone does not fully account for the dominant-negative phenotype, in which heterozygous cells containing structurally intact, cAMP-responsive wild-type regulatory subunits nevertheless fail to sustain effective cAMP-PKA signalling. If reduced cAMP sensitivity were the only abnormality, stronger upstream stimulation would be expected to at least partially restore PKA activity by mass action. However, patients with PRKAR1A-associated ACRDYS1 display elevated circulating PTH together with preserved or even increased urinary cAMP responses to hormone challenge, indicating intact receptors and cAMP generation in vivo [14, 42, 47]. These observations argue that cAMP production is not the limiting step and instead point to dysregulated PKA holoenzyme behaviour as the principal defect. This section therefore develops a unifying framework in which mutant RIα-containing PKA holoenzymes retain catalytic subunits following cAMP stimulation, exerting a dominant-negative effect on PKA population dynamics [11–13, 35].

**Fig. 5:**
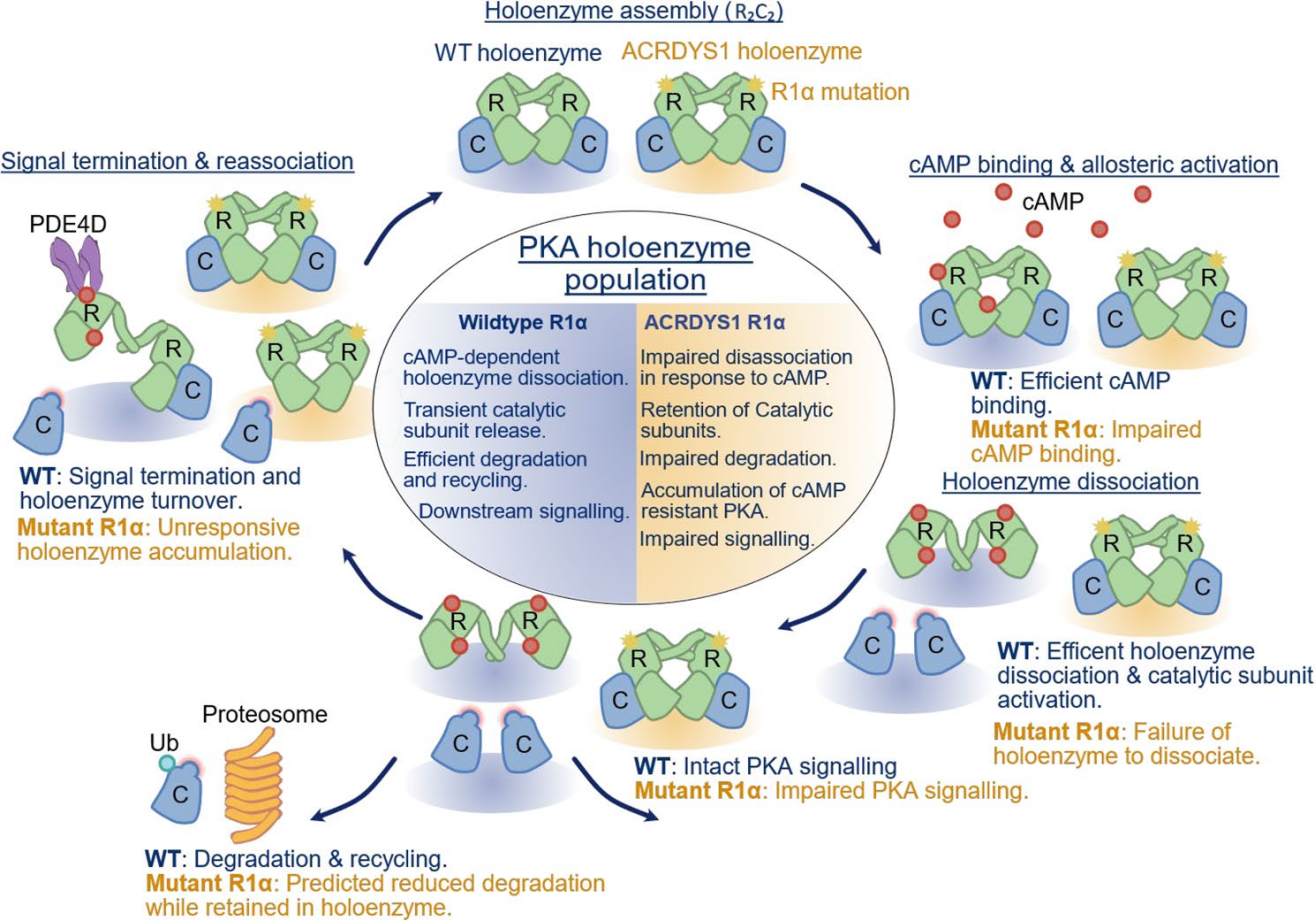
Dual mechanism of impaired RIα activation and catalytic subunit retention in ACRDYS1. Conceptual model illustrating the PKA activation cycle and its disruption by ACRDYS1-associated PRKAR1A mutations. In the wild-type (blue) pathway, RIα regulatory subunits assemble with catalytic subunits to form inactive R₂C₂ holoenzymes, bind cAMP efficiently, undergo allosteric mediated conformational changes and disinhibit to activate catalytic subunits. This enables transient downstream signalling, followed by signal termination, reassociation and regulated holoenzyme turnover. In contrast, ACRDYS1-associated mutant RIα (orange) assembles normally into holoenzymes but exhibits abnormal cAMP binding and defective allosteric activation. As a result, holoenzymes dissociate inefficiently, leading to reduced catalytic subunit activation, attenuated PKA signalling and accumulation of activation-resistant holoenzyme complexes

In this framework, impaired allosteric activation is compounded by altered holoenzyme cycling, together reducing the availability, turnover, and effective deployment of catalytic subunits and creating a hypomorphic signalling state that cannot be rescued by increased upstream stimulation [11–13]. Figure 5 illustrates this proposed retention model, reframing ACRDYS1 as a disorder of altered PKA holoenzyme population dynamics rather than a simple defect in cAMP sensing alone. In the wild-type cycle, RIα regulatory subunits assemble with catalytic subunits to form inactive R₂C₂ holoenzymes that respond to transient cAMP elevations by undergoing coordinated allosteric rearrangements, promoting efficient catalytic subunit disinhibition and robust phosphorylation of downstream substrates (2, 4, 17) (Fig. 5). Following signal propagation, reassociation of regulatory and catalytic subunits, coupled to regulated protein turnover, restores the basal state and preserves responsiveness to subsequent stimuli [18, 54, 55]. In contrast, in ACRDYS1, mutant RIα assembles normally into holoenzymes but fails to support efficient catalytic subunit disinhibition or downstream PKA substrate phosphorylation, resulting in blunted kinase activation despite adequate cAMP levels [11–13, 38] (Fig. 5). A key biochemical feature of the system is the marked excess of regulatory over catalytic subunits, with an approximate R: C ratio of 17:1, which makes cellular catalytic activity intrinsically dependent on holoenzyme dissociation [49]. When dissociation is inefficient, a substantial fraction of the catalytic pool becomes trapped in inactive complexes, perturbing catalytic subunit availability not only within mutant holoenzymes but also at the level of the broader PKA population [18, 27, 28]. In wild-type cells, activation of catalytic subunits enables substrate phosphorylation and simultaneously exposes free kinase to quality-control pathways that govern degradation, recycling, and spatial redistribution, thereby shaping both the intensity and duration of PKA signalling [54, 55]. By contrast, persistent regulatory-catalytic association in mutant RIα-containing holoenzymes would be expected to shield catalytic subunits from clearance mechanisms that preferentially act on free kinase, while also limiting their reincorporation into newly formed, fully responsive holoenzyme pools [54, 55] (Fig. 5). The resulting accumulation of long-lived, activation-resistant complexes offers a mechanistically coherent explanation for the hormone resistance observed in ACRDYS1 patients [35, 39].

Functionally, this altered population constraint is that impaired dissociation has effects extending beyond a single activation event, reshaping the kinetics of cAMP-PKA signalling over time. Experimental studies in heterozygous systems show that mutant RIα suppresses population-level cAMP-PKA activation kinetics even in the presence of wild-type regulatory subunits, flattening dose–response curves and reducing maximal activation [12, 35, 45]. This behaviour indicates that mutant-containing holoenzymes can dominate signalling output across the PKA population, rather than acting solely through reduced responsiveness of individual complexes [35, 49]. Building on these observations, this model proposes that ACRDYS1 reflects a global shift in PKA holoenzyme population dynamics, in which mutant RIα-containing complexes act as kinetic traps that retain catalytic subunits, accumulate as long-lived activation-resistant species, and progressively reduce the pool of catalytically competent kinase [11–13, 49]. In this conceptual framework, hormone resistance arises not from a failure to generate cAMP but from an emergent defect in decoding cAMP signals across the holoenzyme ensemble, providing a testable basis for future quantitative modelling and live-cell studies of catalytic subunit turnover, compartmentalisation, and redistribution during repeated physiological stimulation [25, 26].

### The specificity of ACRDYS1-associated PRKAR1A mutations

Although PRKAR1A is implicated in more than one human disorder, the mutations that cause ACRDYS1 represent a highly specific and mechanistically coherent set of variants [7–9]. Virtually all ACRDYS1-associated variants are missense substitutions, with the notable exception of rare truncation variants located at the extreme C-terminus of RIα. These pathogenic variants cluster within the cyclic nucleotide-binding domains, where they disrupt cAMP affinity, pocket geometry, or the allosteric transitions required for catalytic subunit disinhibition [11, 12]. Importantly, these mutations preserve overall protein expression and holoenzyme assembly, yet selectively impair the activation step of the PKA cycle [11, 12]. This produces a distinctive biochemical signature in which RIα is present but responds poorly to physiological cAMP concentrations, resulting in activation-resistant holoenzyme populations and a hypomorphic PKA signalling state [11–13].

The restricted occurrence of truncation variants in ACRDYS1 reflects strong positional constraints within PRKAR1A [7–9]. In contrast to Carney complex, where truncating variants are distributed throughout the gene [6, 10, 15], ACRDYS1-associated truncations occur only near the C-terminus and result in minimal loss of the RIα protein [7, 11, 39]. For example, the p.Arg368X variant removes fewer than 14 amino acids, and expression of the truncated protein has been experimentally confirmed, indicating that the transcript escapes nonsense-mediated decay [11, 39]. Truncations occurring further upstream instead destabilise the transcript and lead to loss of RIα expression, precluding a dominant-negative mechanism and favouring a haploinsufficiency phenotype observed in Carney Complex [6, 10, 15]. The absence of such upstream truncations in ACRDYS1 therefore likely reflects a form of survivor bias, whereby only variants compatible with stable expression and impaired cAMP responsiveness manifest as disease [6, 11, 39].

The specificity of this mutational pattern becomes clearer when contrasted with the variant spectrum associated with Carney complex, a disorder also linked to PRKAR1A but driven by a fundamentally different molecular mechanism [15, 31] (Table 2). In Carney complex, the majority of pathological alleles are protein-truncating or destabilising mutations that reduce RIα abundance through haploinsufficiency [15, 31]. This loss of regulatory subunit availability decreases restraint on catalytic subunits, leading to increased basal PKA activity [15, 31]. The pathological mechanism is therefore not impaired cAMP responsiveness, but reduced inhibitory buffering, producing a signalling state opposite to that seen in ACRDYS1 [15, 31] (Table 2). This comparison underscores an important mechanistic principle: the disease outcome of a PRKAR1A mutation depends not merely on whether RIα is altered, but on which specific structural or regulatory feature is perturbed [6, 15]. Missense variants that distort the cAMP-binding domains produce ACRDYS1 by compromising allosteric activation while preserving holoenzyme assembly [11, 12]. In contrast, variants that disrupt RIα stability or expression predispose to Carney complex by diminishing the availability of regulatory subunits required to restrain catalytic activity [15, 31] (Table 2).

**Table 2:** Distinct molecular mechanisms of PRKAR1A-associated disease: comparison of ACRDYS1 and Carney complex. Summary of mutation classes, molecular consequences and signalling outcomes associated with ACRDYS1 and Carney complex, based on biochemical, structural and clinical studies of PRKAR1A variants [7–9, 11–13, 15, 31]

Feature	Acrodysostosis type 1 (ACRDYS1)	Carney complex (CNC)
Typical mutation type	Missense variants and truncating.	Truncating, frameshift, splice-site or destabilising variants.
Mutation localisation within RIα	Cyclic nucleotide-binding domains (CNB-A, CNB-B) and C-terminal capping region.	Distributed across whole gene.
Effect on RIα protein abundance	Near-normal expression.	Instances of reduced expression due to haploinsufficiency.
Holoenzyme assembly	Preserved R₂C₂ holoenzyme formation.	Reduced regulatory subunit availability limits holoenzyme formation.
cAMP binding and activation	Impaired cAMP binding or defective allosteric transitions.	Largely intact in remaining Riα.
Catalytic subunit disinhibition	Attenuated or incomplete disinhibition in response to cAMP.	Increased basal disinhibition due to reduced regulatory restraint.
Basal PKA activity	Reduced.	Elevated.
Stimulated PKA responsiveness	Blunted or right-shifted dose-response.	Exaggerated or constitutive.
Primary signalling defect	Defective regulatory activation (hypomorphic signalling).	Loss of inhibitory buffering (hyperactive signalling).
Clinical signalling consequence	Hormone resistance, impaired developmental signalling.	Hormone hypersensitivity, tumour predisposition.
Representative phenotypes	Skeletal dysplasia, endocrine resistance.	Cardiac myxomas, endocrine tumours, skin pigmentation.

Thus, the two disorders highlight the dual vulnerabilities of the PKA system, with ACRDYS1 revealing the consequences of defective regulatory activation and Carney complex illustrating the effects of reduced regulatory abundance [6, 15]. The fact that distinct classes of PRKAR1A mutations produce such different signalling states and clinical phenotypes emphasises how precisely RIα must be tuned, with both its quantity and its signal responsiveness required to maintain appropriate amplitude and timing of PKA signalling during development [2, 6].

### Tissue-specific consequences of impaired RIα-dependent cAMP-PKA signalling

#### Mechanisms underlying growth plate abnormalities in ACRDYS1

**Fig. 6:**
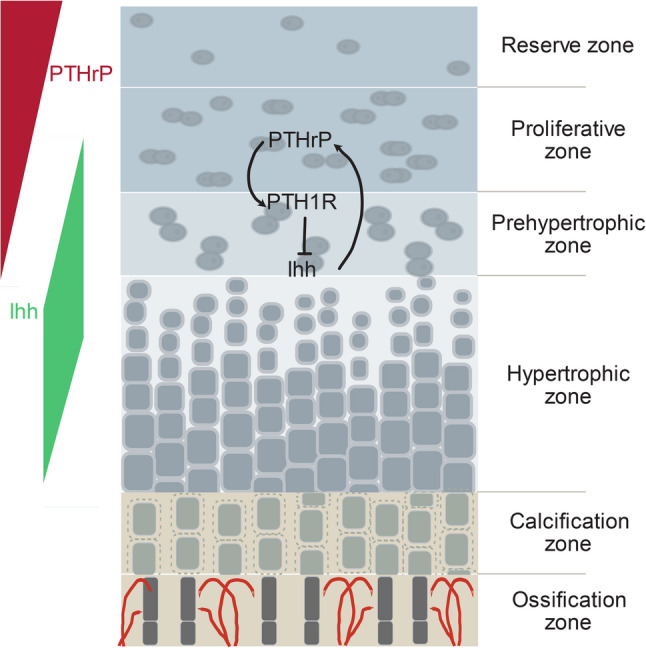
Organisation of the growth plate and the PTHrP–Ihh negative feedback loop regulating chondrocyte maturation. Schematic of the longitudinal growth plate showing the reserve, proliferative, prehypertrophic, hypertrophic, calcification and ossification zones. Cell morphology is illustrated as it matures through each of the zones of the growth plate. Parathyroid hormone–related peptide (PTHrP) is shown acting on PTH1R-expressing chondrocytes in the proliferative and prehypertrophic zones. Indian hedgehog (Ihh) is expressed by prehypertrophic chondrocytes and signals towards the upper growth plate, forming a negative feedback loop with PTHrP

The growth plate is one of the tissues most profoundly affected in ACRDYS1, reflecting its strong reliance on cAMP-dependent PKA signalling to coordinate chondrocyte proliferation and differentiation [7–9, 39]. RIα is the predominant regulatory subunit expressed in proliferative and prehypertrophic chondrocytes, where PKA integrates hormonal and morphogen-derived cues to regulate the balance between columnar proliferation and hypertrophic maturation [6, 39]. Impaired activation of RIα therefore has direct consequences for the spatiotemporal organisation of growth plate maturation [11, 12, 39]. Within this system, the parathyroid hormone 1 receptor is the principal Gs-coupled receptor driving cAMP production in proliferative chondrocytes [42, 47] (Fig. 6). Activation of PTH1R by parathyroid hormone-related peptide, produced in the periarticular region, generates local cAMP signals that must be decoded rapidly and efficiently by RIα-containing holoenzymes [42, 47] (Fig. 6). The resulting PKA activity promotes phosphorylation of CREB and related transcriptional regulators that suppress hypertrophic differentiation and maintain chondrocytes in a proliferative state [23, 31] (Fig. 6). The magnitude and temporal fidelity of RIα activation therefore define the window over which proliferation is sustained before entry into hypertrophy [2, 27]. This RIα-dependent signalling constitutes a core component of the Ihh–PTHrP feedback loop that coordinates longitudinal bone growth [56, 57] (Fig. 6). Prehypertrophic chondrocytes express Indian hedgehog, which signals to the perichondrium to induce PTHrP expression, reinforcing proliferative identity in neighbouring chondrocytes [56, 57]. As proliferating chondrocytes move away from the periarticular source of PTHrP, local ligand concentrations decline, RIα-dependent PKA activation falls and hypertrophic differentiation proceeds [56, 57]. Spatial patterning of the growth plate thus emerges as a functional readout of RIα-mediated PKA signalling thresholds [39, 56].

As a consequence of PRKAR1A mutations, PTH1R stimulation produces attenuated PKA activity that is insufficient to sustain CREB-dependent transcriptional programmes required to maintain chondrocytes in the proliferative state [11, 12, 23]. This reduction in signalling output shortens the duration over which PKA activity remains above the threshold required to sustain the proliferative state, leading to premature transition of proliferative chondrocytes into the hypertrophic lineage even in the presence of PTHrP [39]. Genetic evidence from in vivo models supports this mechanism: knock-in mice harbouring the recurrent R368X PRKAR1A mutation exhibit premature chondrocyte hypertrophy, compression of the proliferative zone and advanced bone age, demonstrating that impaired RIα activation alone is sufficient to disrupt growth plate patterning [39]. These architectural changes recapitulate the cardinal skeletal features observed in ACRDYS1, including shortened long bones, epiphyseal dysplasia and accelerated skeletal maturation [7, 9, 39].

The exceptional sensitivity of the growth plate to PRKAR1A mutations reflects its heavy reliance on RIα-containing holoenzymes and its limited capacity to compensate through RII–AKAP-anchored PKA signalling [2, 6, 25]. Unlike tissues with prominent type II PKA microdomains, proliferative chondrocytes depend on fast, low-threshold RIα-mediated responses to transient cAMP signals [2, 27, 39]. This tissue-specific dependence explains why skeletal abnormalities are among the earliest and most penetrant manifestations of ACRDYS1 [7–9].

### Extension of the endochondral growth defect to craniofacial structures

Craniofacial abnormalities in ACRDYS1, including midface hypoplasia, depressed nasal bridge and brachycephaly, can be interpreted as an extension of the same endochondral growth defect [7, 8, 41]. Clinical and radiological evidence indicate that these features frequently co-occur with advanced bone age and cone-shaped epiphyses, findings that specifically reflect dysregulated endochondral cartilage growth and maturation, thereby supporting disruption of cartilage-based growth rather than isolated defects in intramembranous ossification [41, 42]. Consistent with this interpretation, PRKAR1A R368X heterozygous knock-in mice display quantifiable craniofacial dysostosis on µCT analysis, including reduced cranial dimensions and altered skull base morphology [39].

Craniofacial growth relies on specialised cartilage growth centres, particularly the cranial base synchondroses, which coordinate midfacial elongation through precisely timed chondrocyte proliferation and hypertrophic differentiation [45, 46]. These structures place stringent demands on signal integration and threshold sensing during development [45]. Given that many of the pathways governing synchondrosis maturation converge on cAMP-dependent PKA signalling, reduced RIα responsiveness is predicted to disproportionately affect craniofacial cartilage dynamics [11, 12, 45]. Although direct molecular analysis of cranial base synchondroses in ACRDYS1 models remains limited, the parallel disruption of appendicular and craniofacial cartilage growth supports a shared mechanism rooted in impaired RIα-dependent signal decoding [39].

### Endocrine resistance as a defect in cAMP signal decoding downstream of receptor activation

Endocrine resistance is a defining clinical feature of ACRDYS1 and is most consistently observed for parathyroid hormone and thyroid-stimulating hormone [7, 8, 10]. In contrast to disorders caused by impaired hormone production or receptor dysfunction, endocrine resistance in ACRDYS1 reflects failure to decode hormone-induced cAMP signals into appropriate intracellular and transcriptional responses within target tissues [7, 12].

Clinical biochemical data provide localisation of the defect within the signalling pathway. Specifically, individuals with ACRDYS1 exhibit elevated circulating PTH concentrations together with normal or low serum calcium and phosphate levels, a pattern that reflects renal PTH resistance in which hormone-induced cAMP generation is intact but fails to activate downstream PKA-dependent effector pathways [7, 8]. Consistent with this interpretation, urinary cAMP levels are frequently normal or elevated, demonstrating preserved receptor coupling, Gsα function and adenylyl cyclase activity [7, 8, 10]. Together, these biochemical findings localise the lesion downstream of cAMP generation, at the level of cAMP-dependent signal decoding [12].

A similar pattern is observed in the thyroid axis, where elevated TSH concentrations coexist with normal or mildly reduced thyroid hormone levels [7, 8]. In thyroid follicular cells, hormone synthesis depends on sustained PKA-dependent transcriptional programmes that regulate expression of genes required for iodide uptake, organification and hormone biosynthesis, rather than on transient cytosolic phosphorylation events [23, 27]. Attenuation of RIα-mediated PKA activation amplitude or duration is therefore sufficient to impair thyroid hormone output without disrupting upstream receptor signalling or cAMP generation [23, 27]. Endocrine epithelia such as renal proximal tubule and thyroid follicular cells thus rely on RIα-dependent PKA activity as a transcriptional gate that must be exceeded and maintained to drive CREB-dependent gene expression, rather than as a brief, acute kinase response [23, 27]. Flattening of the PKA activation dynamic range decouples hormone-induced cAMP accumulation from downstream transcriptional activation, producing multihormone resistance without global disruption of signalling pathways [12]. This mechanism clearly distinguishes ACRDYS1 from pseudohypoparathyroidism type 1a, in which resistance originates upstream at the level of Gsα function [14].

### Regulatory subunit redundancy and selective tissue vulnerability

Neurodevelopmental and cognitive features have been reported in a subset of individuals with ACRDYS1, but penetrance is variable and manifestations are generally milder than those affecting skeletal or endocrine tissues [7, 8, 10]. This relative sparing of the nervous system is consistent with the distinctive organisation of cAMP–PKA signalling in neuronal lineages, where dependence on RIα is limited and substantial functional redundancy exists among PKA regulatory subunits.

Unlike growth plate cartilage or endocrine epithelia, neuronal cAMP-PKA signalling is distributed across multiple regulatory subunits and anchoring configurations. PRKAR1B (RIβ) is highly enriched in many neuronal populations, particularly within cortical and hippocampal circuits, where it supports synaptic maturation, plasticity and activity-dependent transcription [5, 22, 31]. In parallel, RIIα- and RIIβ-containing holoenzymes, tightly scaffolded by AKAPs, dominate many neuronal microdomains and mediate compartmentalised PKA signalling at synapses and dendritic spines [3, 25]. As a result, neuronal signalling networks do not rely exclusively on RIα-dependent, low-threshold decoding of transient cAMP signals [22, 26]. Because ACRDYS1 arises specifically from pathological mutations in PRKAR1A, impairment is largely confined to RIα-containing holoenzymes [7, 12, 38, 45]. In neuronal tissues where PRKAR1B and PRKAR2A/2B are expressed at higher levels than PRKAR1A [22, 23], cAMP signalling can still proceed through these alternative regulatory subunits, so that loss of RIα responsiveness caused by PRKAR1A mutation may have a limited impact on overall PKA activity. This redundancy may therefore preserve core developmental processes such as neuronal survival, migration and circuit formation that depend on cAMP-PKA signalling in the nervous system [5, 31, 32]. Consequently, regulatory subunit redundancy provides a mechanistic explanation for why ACRDYS1 does not present with a uniform or severe neurodevelopmental phenotype despite the central role of cAMP–PKA signalling in neuronal development.

A similar logic applies to metabolic tissues. In adipose tissue, liver and central metabolic circuits, cAMP–PKA signalling is distributed across multiple regulatory subunits with distinct functional roles. RIα-containing holoenzymes respond rapidly to transient increases in cAMP and mediate acute hormonal responses such as β-adrenergic stimulation of lipolysis and substrate mobilisation [52, 53]. In parallel, RIIβ-containing complexes are highly expressed in metabolic tissues and are typically anchored to AKAP scaffolds that organise signalling within defined subcellular microdomains [52, 53]. Because ACRDYS1 arises from mutations in PRKAR1A, signalling defects are expected to affect RIα-containing holoenzymes most directly. However, the presence of alternative regulatory subunits, particularly RIIβ, may allow partial preservation of cAMP–PKA signalling. This redundancy likely contributes to the variable and generally mild metabolic phenotypes observed in ACRDYS1, reflecting altered signalling dynamics rather than complete disruption of core metabolic pathways [52, 53, 58].

Dental tissues appear to share this vulnerability profile. Odontogenesis depends on sustained transcriptional programmes in ameloblasts and odontoblasts that coordinate matrix secretion, mineralisation and root maturation over extended developmental windows [56, 57]. In these cells, cAMP–PKA signalling contributes to transcriptional regulation of differentiation and matrix production through CREB-dependent pathways [2, 21, 26]. Because ACRDYS1 arises from mutations in PRKAR1A that impair RIα-containing PKA holoenzymes, disruption of RIα-dependent signalling is predicted to reduce the efficiency or duration of these transcriptional programmes. As a result, alternative regulatory subunits may provide limited functional compensation for impaired RIα activation during tooth development. This mechanism is consistent with enamel hypoplasia and shortened roots observed clinically in individuals with ACRDYS1 while leaving early developmental patterning largely intact [7–9, 12].

### Mechanism-informed therapeutic considerations in ACRDYS1

Management of ACRDYS1 is currently supportive and focuses on mitigating downstream endocrine, skeletal and dental manifestations rather than correcting the underlying signalling defect [7–10, 14]. Hormone replacement for hypothyroidism, management of mineral homeostasis in parathyroid hormone resistance, and orthopaedic or orthodontic interventions provide symptomatic benefit but do not restore normal cAMP–PKA signal decoding at the level of RIα [7–9, 14]. The limited efficacy of growth hormone therapy in improving final height further illustrates this principle. Although endocrine stimulation is increased, the intracellular signalling machinery required to decode hormonal cues remains impaired. Consequently, ACRDYS1 is not primarily a deficiency of endocrine stimulation but a disorder of intracellular signal transduction downstream of hormone receptors [7, 9, 10].

Biochemical assays using purified recombinant RIα proteins, together with cell-based studies in patient-derived fibroblasts and engineered cell lines, indicate that most mutant RIα proteins are expressed at near-normal levels and assemble efficiently into holoenzymes [11–13]. However, PRKAR1A variants exhibit right-shifted cAMP dose–response curves, reduced maximal activation and impaired allosteric coupling between cyclic-nucleotide binding domains [11–13]. These findings indicate that ACRDYS1 arises from impaired cAMP-dependent activation of RIα rather than reduced protein abundance or defective holoenzyme assembly. In this context, increasing upstream hormonal stimulation cannot fully compensate for defective RIα-mediated signal decoding. Therapeutic strategies aimed at restoring or enhancing RIα responsiveness to cAMP may therefore represent a more rational conceptual approach than further amplification of endocrine stimulation [11–13].

One potential therapeutic strategy involves the use of cyclic-nucleotide analogues or small molecules designed to stabilise the active conformation of RIα or enhance cAMP binding to mutant cyclic-nucleotide binding domains. Structural analyses of recurrent ACRDYS1 mutations reveal distortions in the phosphate-binding cassette and destabilisation of the C-terminal capping element, both of which increase the energetic barrier for catalytic subunit disinhibition [11, 12]. Consistent with this mechanism, partial activation of certain mutant holoenzymes can be achieved under supraphysiological cAMP conditions, suggesting that pharmacological sensitisation strategies could, in principle, increase signalling output. However, therapeutic translation is constrained by the challenge of restoring RIα activation while preserving the spatial and temporal compartmentalisation that normally governs cAMP–PKA signalling. Global elevation of cAMP or non-selective sensitisation of PKA risks disrupting microdomain-restricted signalling networks and the narrow physiological window separating insufficient and excessive PKA activity [2, 6, 15].

A second conceptual strategy arises from evidence that activation-resistant RIα variants impose a dominant constraint on catalytic subunits by prolonging regulatory–catalytic association [12, 13]. Therapeutic approaches that destabilise mutant-specific regulatory–catalytic interfaces, or promote disinhibition of catalytic subunits from non-productive holoenzyme complexes, could increase the effective pool of signalling-competent kinase without altering upstream cAMP production. In parallel, allele-selective transcript suppression using antisense or other RNA-based strategies could theoretically rebalance regulatory subunit composition in favour of wild-type RIα. However, such approaches would require exceptional specificity to avoid unintended shifts toward excessive PKA activation [6, 15] and therefore remain highly speculative.

Gene-based therapeutic approaches represent a potential long-term strategy for addressing the underlying molecular defect in ACRDYS1. In principle, precise correction of pathogenic PRKAR1A variants through base editing or prime editing could restore wild-type RIα structure and re-establish normal cAMP-dependent activation of PKA. Because most ACRDYS1-associated variants are missense mutations that impair allosteric transitions within the cyclic-nucleotide binding domains rather than abolishing protein expression, targeted repair of the mutant allele offers a theoretically direct route to restoring physiological signalling dynamics [11–13, 38, 45]. A major challenge arises from the extreme dosage sensitivity of the cAMP–PKA signalling system. Evidence from PRKAR1A haploinsufficiency in Carney complex demonstrates that even modest reductions in RIα expression produce substantial increases in basal PKA activity [6, 15]. Together with the impaired activation phenotype observed in ACRDYS1 [11–13, 45], these disorders illustrate that both insufficient and excessive RIα function can disrupt signalling homeostasis [6, 11–13, 15]. Gene-based interventions would therefore need to restore regulatory subunit function within a very narrow physiological range, requiring precise spatial and quantitative control of PRKAR1A expression across affected tissues. For these reasons, allele-selective strategies may represent a more mechanistically coherent approach. Selective suppression or correction of the mutant allele; using antisense oligonucleotides, RNA-based silencing, or targeted editing; could reduce production of activation-resistant RIα while preserving endogenous expression of the wild-type allele [6, 15]. In principle, such approaches could rebalance regulatory subunit composition and improve cAMP-dependent activation without driving excessive PKA activity. Nevertheless, achieving the required specificity while maintaining appropriate tissue distribution and signalling balance remains a technical challenge.

Overall, mechanistic insights into RIα dysfunction in ACRDYS1 provide a framework for rational therapeutic exploration but also highlight the inherent difficulty of targeting a ubiquitously expressed second-messenger pathway. Effective intervention is likely to require strategies that restore local signalling dynamics within vulnerable tissues rather than globally amplifying cAMP–PKA activity. Continued integration of structural biology, patient-derived cellular models and tissue-specific signalling analyses will be essential to translate these concepts into safe and effective therapies.

## Conclusion

ACRDYS1 illustrates how selective disruption of a single regulatory node within the cAMP–PKA pathway can give rise to a complex multisystem developmental phenotype. Disease-associated PRKAR1A mutations cluster within the cyclic nucleotide–binding domains, mediate interdomain communication, secure the active conformation of the protein, and C-terminal capping region of RIα and converge on a shared biochemical defect: impaired cAMP-driven allosteric conformational change that limits catalytic subunit disinhibition despite preserved holoenzyme assembly [11, 12]. This produces a hypomorphic type I PKA signalling state in which physiological cAMP elevations are decoded inefficiently and, in heterozygous contexts, are further constrained by persistence of activation-resistant holoenzyme populations that retain catalytic subunits and flatten population-level activation dynamics [12, 35]. The tissue specificity of ACRDYS1 reflects differences in cAMP microdomain organisation and regulatory subunit usage, with tissues that rely heavily on RIα-mediated decoding of transient cAMP signals, including growth plate cartilage and endocrine epithelia, being most consistently affected, while contexts dominated by alternative regulatory subunits or AKAP-anchored type II signalling exhibit partial buffering [2, 6]. This framework also clarifies the distinction from PRKAR1A-related Carney complex, which arises from reduced RIα abundance and basal PKA hyperactivity, in contrast to the preserved expression but defective activation that defines ACRDYS1 [6, 15]. Together, this discussion emphasises both the specificity and the therapeutic challenge of RIα dysfunction, indicating that effective intervention will require restoration of appropriate local cAMP-PKA signalling dynamics rather than global pathway amplification [2, 11, 12, 39].

## Data Availability

No datasets were generated or analysed during the current study.

## Abbreviations

ACRDYS1: Acrodysostosis type 1
AC: Adenylate cyclase
ACTH: Adrenocorticotropic hormone
AKAP: A–kinase anchoring protein
ARC: Activity–regulated cytoskeleton–associated protein
AR: Androgen receptor
ASO: Antisense oligonucleotide
BDNF: Brain–derived neurotrophic factor
BMP: Bone morphogenetic protein
cAMP: Cyclic adenosine monophosphate
CNC: Carney complex
CNB: Cyclic nucleotide–binding (domain)
CREB: cAMP response element–binding protein
D/D: Dimerisation/docking (domain)
DNA: Deoxyribonucleic acid
GH: Growth hormone
GPCR: G protein–coupled receptor
GRK: G protein–coupled receptor kinase
HSL: Hormone–sensitive lipase
IGF: 1–Insulin–like growth factor 1
IHH: Indian hedgehog
IS: Inhibitory site (of regulatory subunits)
LH: Luteinising hormone
LKB1: Liver kinase B1 (also known as STK11)
NMD: Nonsense–mediated decay
PBC: Phosphate–binding cassette
PDE: Phosphodiesterase
PKA: Protein kinase A (cAMP–dependent protein kinase)
PPNAD: Primary pigmented nodular adrenocortical disease
PTH: Parathyroid hormone
PTH1R: Parathyroid hormone 1 receptor
PTHrP: Parathyroid hormone–related peptide
R (subunit): Regulatory subunit of PKA
R₂C₂: Tetrameric PKA holoenzyme containing two regulatory and two catalytic subunits
RIα: Type Iα regulatory subunit of PKA (encoded by PRKAR1A)
RIβ: Type Iβ regulatory subunit of PKA
RIIα: Type IIα regulatory subunit of PKA
RIIβ: Type IIβ regulatory subunit of PKA
RNAi: RNA interference
Smad: Mothers against decapentaplegic homolog (BMP/TGF–βsignalling mediators)
STPK: Serine/threonine protein kinase
TGF: β–Transforming growth factor beta
TSH: Thyroid–stimulating hormone
VSMC: Vascular smooth muscle cell
WT: Wild type
